# How mental health status and attitudes toward mental health shape AI Acceptance in psychosocial care: a cross-sectional analysis

**DOI:** 10.1186/s40359-025-02954-z

**Published:** 2025-06-06

**Authors:** Birthe Fritz, Lena Eppelmann, Annika Edelmann, Sonja Rohrmann, Michèle Wessa

**Affiliations:** 1 Department of Clinical Psychology and Neuropsychology, Johannes Gutenberg-University Mainz, Wallstraße 3, Mainz, 55122 Germany; 2 Department of Differential Psychology and Psychological Assessment, Johann Wolfgang Goethe University, Institute for Psychology, Campus Westend | PEG Building, Frankfurt Am Main, 60629 Germany; 3 Leibniz-Institute for Resilience Research (LIR) gGmbH, Wallstraße 7, Mainz, 55131 Germany; 4 Department of Neuropsychology and Psychological Resilience Research, Central Institute of Mental Health, Heidelberg University, Mannheim, J5, 68159 Germany; 5 DKFZ Hector Cancer Institute at the University Medical Center Mannheim, Mannheim, Germany; 6 German Cancer Research Centre (DKFZ), Division of Cancer Survivorship and Psychological Resilience, Im Neuenheimer Feld 280, Heidelberg, 69120 Germany

**Keywords:** Artificial intelligence, Psychological distress, Mobile applications, Mental health, Health literacy, Personality

## Abstract

**Introduction:**

Artificial Intelligence (AI) has become part of our everyday lives and is also increasingly applied in psychosocial healthcare as it can enhance it, make it more accessible, and reduce barriers for help seeking. User behaviour and readiness for AI can be predicted by various factors, such as perceived usefulness (PU) of AI, personality traits and mental health-related variables. Investigating these factors is essential for understanding user acceptance and the future use of AI tools in mental health. This study examines the individual factors that influence the PU of AI in mental health care. In addition, it examines how PU of AI affects the use of mental health apps. For ethical and practical reasons, these apps were considered independently of their AI integration, aiming to support the development of AI-driven mental health applications.

**Method:**

In a German-speaking convenience sample *N* = 302 participants socio-demographic information, personality factors, mental health status, mental health literacy, and various aspects concerning the integration of AI into psychosocial care (PU, AI awareness, digital skills, app use in general) were assessed. Two linear, stepwise regression analyses were conducted, with PU of AI and the participants’ use of mental health apps in general as dependent variables, respectively, and the above-mentioned variables as predictors. Profession, gender, own experience with mental impairments, AI awareness and digital skills were included as covariates. Finally, we performed two moderation analyses to investigate mental health problems and psychological distress as moderators for the relationship between PU and frequency of mental health-related app use—irrespective of AI integration—with working field and digital capabilities as covariates.

**Results:**

Higher openness, pessimism and conscientiousness predicted lower PU, whereas higher agreeableness, lower levels of stigma and social distance predicted higher PU. The covariates psychological/ pedagogical training, digital capabilities and experience had a significant influence on PU. Higher frequency of app use in general was predicted by better digital capabilities, higher psychological distress, and more help seeking behaviour. The relationship between PU and the overall use of mental health apps was moderated by psychological distress but not by mental health problems.

**Discussion:**

Our study identified individual factors influencing PU for integrating AI into psychosocial care and the frequency of using mental health apps—irrespective of AI integration—and thereby underlines the necessity to tailor AI interventions in psychosocial care to individual needs, personality, and abilities of users to enhance their acceptance and effectiveness.

**Supplementary Information:**

The online version contains supplementary material available at 10.1186/s40359-025-02954-z.

## Background

AI-based applications have not only found their way into our everyday lives but are also increasingly being used in the field of mental health, e.g. for self-help, prevention and psychotherapeutic intervention [1, 2]. From the perspective of mental health professionals, technologies that use AI could support, assist, and partially or completely take over various tasks (e.g., with respect to psychoeducation or diagnostic processes), yet, of course, ethical issues have to be considered. From the perspective of mentally burdened persons, AI tools appear to enable low-threshold access, being available at any time anywhere, and reduce barriers for help-seeking due to fewer concerns to address personal issues [3–5]. The availability of and interest in AI-supported applications regarding mental health is constantly rising with applications ranging from chatbots (e.g. Woebot or MyCompass) [6, 7] to tools offering support from mood-, habit- and thought-tracking over relaxation to cognitive-behavioural therapy interventions [8, 9]. In the mental health sector, AI is being used in different ways, for example, through natural language processing (NLP), which means that chatbots engage in human-like conversations based on the analysis of users'text inputs; through machine learning (ML) algorithms, analysing user behaviour and inputs to offer personalised content or interventions; and, more recently, through general AI and deep learning (DL), enabling them to generate content such as text, images, music or videos, or to perform more complex tasks [1, 7, 9, 10].

Research is increasingly looking at how the use of digital health applications affects psychological processes such as thinking, feeling and acting, and thus how they influence people's health and well-being (referred to as cyber health psychology; [11, 12]) and a growing body of evidence that at least some of the existing mental health applications are effective in improving mental health [7, 13].

To date, there is less research on the predictors that promote the use of digital health applications and the factors that prevent people from using them, although the acceptance of AI-supported technologies varies greatly inter-individually [14]. Therefore, it seems reasonable to apply general models of user acceptance of AI technologies, including the widely used extended technology acceptance model (TAM; [15, 16]), to the sector of digital and AI-based mental health applications. Indeed, the importance of TAM for use in healthcare has previously been emphasized [17, 18].

The aim of this study was to identify key factors that predict the PU of AI in psychosocial care and the frequency of mental health app use. For ethical and practical reasons app use was considered independently of AI integration to support the development of future AI-driven mental health tools. Furthermore, the study investigated whether psychological distress and mental health problems moderate the relationship between PU and frequency of app use.

## Literature review

### Technology Acceptance Model (TAM)

**Fig. 1:**
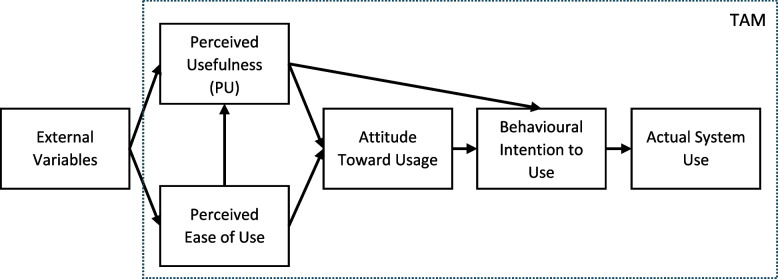
Illustration of the Technology Acceptance Model (TAM). Notes. Dotted line indicates the TAM, whereas external variables are included by TAM2

The TAM (see Fig. 1) was derived from the theory of reasoned action (TRA) and can be considered the primary model for predicting the potential acceptance or rejection of technology in general.

It includes two initial factors, 1) perceived usefulness (PU) and 2) perceived ease of use, both contributing to the formation of an attitude, which in turn leads to behaviour intentions, ultimately culminating in actual usage [17, 19, 20]. There is consistent evidence for a significant relationship between PU and behavioural intentions [17], however, although proposed by the TAM and other theories on the acceptance and use of technology (e.g., the Unified Theory of Acceptance and Use of Technology (UTAUT; [21]) results on their relation to the actual use is rather mixed [17]. Some studies even show no relation at all, which indicates a gap between the behavioural intention—which is directly influenced by the PU—and the real use of AI-driven technology [22, 23]. One approach to understand this gap could be to identify predictors of PU as precursor of the behavioural intention, as some of these predictors could also be relevant moderators of the association between intention and the actual behaviour (here: use of AI technologies in health care). In the TAM2, a further elaboration of the TAM by Venkatesh and Davis [24], such predictors have been included as external variables, influencing the perceived ease of use and the PU (see Fig. 1). On an individual level, different external variables have been proposed in the context of the TAM2, for example, personality traits, socio-demographic aspects, and self-efficacy with regard to digital technologies [19, 24]. Further, in order to take greater account of the particularities of digital interventions in the healthcare sector, Kim and Park [25] developed the Health Information Technology Acceptance Model (HITAM) by including personal health factors as for example the perceived susceptibility pursuant to the Health Belief Model [26] and beliefs as defined in the theory of planned behaviour [27]. Accordingly, the HITAM model includes three areas, one focusing on health, a second on information and a third on technology [25, 28]. For identifying individual predictors influencing PU of AI, perceived ease of use and, ultimately, actual use in the context of psychosocial care, the consideration of mental health related variables seems particularly important as has not extensively been investigated to date.

### External variables in the TAM2: Sociodemographic and Personality

Based on the described theoretical models, previous studies investigated the influence of external variables on PU and actual use of AI technologies, in general, and indeed showed a significant influence of several socio-demographic variables. Some studies observed significant gender differences, with men exhibiting higher AI acceptance compared to women [3, 29, 30]. However, Alanzi et al. [31] investigated the influence of socio-demographic factors on AI-based mental health virtual assistant acceptance and found no significant influence of gender on the use of AI technology for mental health support. In summary, gender seems to have an influence on PU, rather than on the current use [31, 32]. In most studies, age emerged as a factor influencing the PU of AI and the app-usage, whether it was AI in general or AI with a psychosocial application. Older individuals tended to express a less favourable attitude towards these new technologies compared to younger counterparts [29, 31–33]. In other studies, however, age showed no significant influence [30, 34]. In the study by Centeno-Martín et al. [30], among sociodemographic variables only a higher income and higher education led to better attitudes towards communicative AI whereas in the study of Lipschitz et al. [32] education had no influence on the interest in mental health apps. Besides education, the profession seems to be particularly relevant for the use of AI in psychotherapy: People working in the healthcare sector tended to reject AI-supported psychotherapy, while people from the technology/engineering sector tended to prefer it [3]. This fits to the finding that computer use in general has an influence on positive attitudes, but not on negative attitudes towards AI [34].

Some studies have already revealed that personality traits—in particular agreeableness and openness—seem to influence PU and the usage of AI-technologies [30, 31, 34], whereas AI anxiety had a negative effect on the intention to use AI [35]. For the BIG-five personality traits (openness, consciousness, extraversion, agreeableness, neuroticism) Stein et al. [36] found that persons with a higher agreeableness are more positive towards AI and Centeno-Martín et al. [30] showed a significant positive influence of openness on attitudes towards AI. In both studies none of the other BIG-five personality traits showed an association on the attitude towards AI. A study on technology adoption in financial institutions in Pakistan [37] and a study on user perceptions of artificial intelligence [38] showed a significant positive impact of optimism on PU.

### External variables in the TAM2: Mental health

Finally, own mental health burden seems to contribute to more interest in mental health apps and usage of those [32, 39–41]. Results regarding previous treatment experience are heterogeneous: In some studies it was neither associated with the likelihood of using digital interventions, irrespective of AI elements [42], nor with attitudes towards AI-based psychotherapy [3]. In contrast, Borghouts et al. [40] showed a positive effect of treatment experience on the use of mental health apps, without participants specifying whether the apps incorporated AI.

In addition to symptoms of mental disorders (e.g. depression, anxiety or obsessive–compulsive disorders), general mental stress burden as shown by, e.g., restlessness, nervousness and exhaustion could affect the PU or the use of digital mental health technologies [43, 44]. Furthermore, aspects like mental health literacy (MHL) could to be influencing factors for mental health app usage [45]. MHL is a constantly evolving concept [46, 47] and includes the ability to recognise mental illness and distress, to seek knowledge and information about it, to know about risk factors, causes, self-treatment options and available professional help, and to have attitudes that encourage appropriate help-seeking [48]. In the present study, MHL has been operationalized as knowledge about mental health, attitudes regarding mental health and help-seeking intentions. To the best of our knowledge, to date, only a few studies investigated the influence of MHL on PU or usage of mental health technology, showing that whereas MHL was only related to service utilization but not mental health app usage, stigma against individuals with psychological disorders and stigma against help‐seeking was related to the usage of mental health apps [e.g. 50]. Another study reported a positive correlation between higher stigma for receiving psychological help and more positive attitudes towards AI-based psychotherapy [3]. Further, some studies have investigated this relationship with respect to health literacy, showing that health literacy has an influence on more positive attitudes towards AI assisted medical consultations [49]. Interestingly, the authors could show that more disease prevention and health promotion literacy raised the positive attitude, and more healthcare literacy decreased the positive attitude towards AI.

### Hypotheses development and research model

**Fig. 2:**
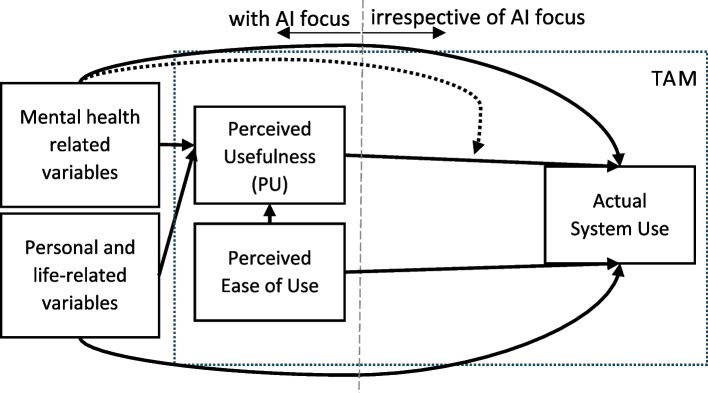
Aspects of the TAM model that are examined in the context of the hypotheses. Notes. Compare Fig. 1 for the structure of the TAM and TAM2; Dotted line indicates moderation analysis (hypothesis 3); only for moderation analysis: inclusion of working area (population, pastoral caregiver and persons with pedagogical/psychological training) and digital competence and AI awareness as covariates; actuals system use = frequency of mental health app usage

As suggested by several technology acceptance models and confirmed by the empirical results delineated above, individual differences in socio-demographic and personality variables influence the perceived usefulness of integrating AI into psychosocial care and the acceptance of mental health apps [e.g. 50]. There is, however, limited research regarding the influence of mental health related variables (e.g. psychological distress, mental health symptoms, mental health literacy) on the PU of integrating AI into mental health care and mental health app usage in general or with AI focus [51, 52]. The mental health status adds an important additional dimension, because individuals may evaluate and use digital solutions for mental health aspects – including or excluding AI-based approaches – not only due to general openness but also due to their knowledge on mental health or their acute need for support. As a systematic review indicated, the current research on digital interventions in mental health focuses primarily on digital tools for interventions and how the mental health status of users changes after the intervention [53] but not on predictors, including mental health-related ones, that influence the PU of AI in psychosocial care or the usage of digital mental healthcare tools (including or not AI elements). The present study therefore adopted a three-step approach, applying the TAM/TAM2 model to mental health technologies. It emphasizes psychological predictors, such as personality and mental health-related factors, that influence the PU and perceived ease of use—key starting points of TAM—and examines the actual use of mental health applications as the model's endpoint (outcome) (see Fig. 2).

In a first step, we investigated which personality and mental health related factors are influencing the PU of integrating AI-based technologies into mental health care and in a second step, how these factors influence actual app usage, irrespective of AI integration. For several reasons, app usage is assessed in general in this study (in line with some previous studies) and not only with a specific focus on AI elements: First, the dissemination and availability of AI-based applications is currently limited in Germany due to, e.g., data protection guidelines and the need to address bias and transparency issues [54–56]. This makes it difficult to collect specific data on the use of AI applications in the field of mental healthcare and underscores the need to investigate more general App usage. Previous research suggests, however, that existing experiences and attitudes shape the evaluation of new technologies [e.g. 57]. Therefore, the consideration of apps independently of their AI integration can be a useful proxy for the investigation of influencing factors and use of new digital technologies in mental health care to develop hypotheses regarding predictors for and the use of specialised technologies—such as AI-based applications—in the field of mental health care and prevention. This, in turn, might also give insights into important caveats for the introduction of such tools. Second, information of AI elements in health care apps gathered from individuals in the general population might not always be valid and reliable as – on average – they might not have enough specialized knowledge to evaluate the extent to which an application uses AI elements. In the present study, we were not able to track user data directly from the smart phone and thus the app itself but had to rely on the self-report of study participants. Accordingly, the present study focuses on all digital apps related to mental health, irrespective of AI integration. We investigated the following hypotheses, for which sociodemographic factors, working area, digital competence, AI awareness, and personal treatment experiences were included as covariates, respectively, to explore the unique influence of the independent variable of interest on the dependent variable beyond the effect of the covariates. Digital competence and AI awareness are understood as competencies or effort required for use and can therefore be regarded as perceived ease of use [19, 20]. The following two explorative questions were investigated:

1. Controlling for the covariates sociodemographic factors, working area, digital competence, AI awareness, and personal treatment experiences, does each of the following independent variables:
  - Mental health status.
  - Attitudes towards mental health.
  - Mental health knowledge.
  - Personality traits.
    contribute significantly to explaining the variance in the PU of AI in psychosocial care (dependent variable)?
2. Controlling for the covariates sociodemographic factors, working area, digital competence, AI awareness, and personal treatment experiences, does each of the following independent variables:
  - Mental health status.
  - Attitudes toward mental health.
  - Mental health knowledge.
  - Personality traits.

contribute significantly to explaining the variance in the frequency of mental health app usage (dependent variable)?

In a third step, the study will combine PU and usage. As demonstrated by the Health Belief Model [58]—one of the earliest frameworks for explaining and predicting health behaviours—the likelihood of engaging in a health behaviour depends on two key factors: the perceived threat of a health issue (e.g., psychological stress or symptoms) and the expected benefit of the preventative measure (in this case, the PU of AI-based applications in mental health) [26, 58, 59]. Accordingly, psychological distress and stress may act as moderating factors that influence both the PU and subsequent usage behaviour. In conclusion, we conducted moderator analyses to investigate the following third explorative question (see Fig. 2), for which working area and digital competence and AI awareness were included as covariates:

3. To what extent do psychological distress and/or mental health problems moderate the relationship between PU of integrating AI into psychosocial care and frequency of mental health app usage?

The present study aims to address another research gap by including a sample from the general population as well as individuals working in mental health care or related fields, such as pastoral care. To date, the acceptance and use of mental health apps for diagnostics and treatment have been considered predominantly and therefore studies mainly included individuals affected by mental health issues [53, 60, 61]. Studying the general population yet can help guide the development of digital health applications specifically designed for use in primary prevention—prior to the onset of illness.

To answer our research questions, we used a non-validated questionnaire assessing the dependent variables ‘PU of integrating AI into psychotherapy’ and the variable ‘digital competence and AI awareness’. We validated this questionnaire and report the respective results in the section of self-report instruments and the Supplement S2.

## Methods

### Procedure

Data collection for the study took place online in Spring 2023 via SoSci Survey (Leiner [62], www.soscisurvey.de). The overall project was a ‘study on MHL in the general population and in specific occupational groups’ in Germany and the present analyses are partial analysis of the overall data and constructs.

Participants were recruited through advertising flyers, by email via mailing lists of the church districts, via social media platforms (Facebook, Instagram), and personal contacts. Accordingly, the sample is a convenience sample. Full participation (approximately 60–70 min) was compensated with €15 provided by Johannes Gutenberg University (JGU) Mainz, optionally payable or donatable to a charity organization funding and supporting projects related to mental health (Aktionsbündnis Seelische Gesundheit). Inclusion criteria for the study were a minimum age of 18 years and proficient German language skills. The study adhered to anonymity and data protection regulations as well as to the Declaration of Helsinki [63]. The Ethics Commission of JGU Mainz reviewed and approved the study as ethically unobjectionable (Application 2022-JGU-psychEK-S041). Participants were informed about the nature and the procedure of the study as well as data protection guidelines and gave written informed consent before participating.

### Sample description

From initially *N* = 542 individuals starting the online questionnaire, *n* = 302 individuals were included in the analysis; *n* = 240 participants did not complete all questionnaires and were therefore excluded from analyses. The final sample consisted of *n* = 137 pastoral caregivers, *n* = 114 individuals from the general population, and *n* = 51 individuals with basic psychological-pedagogical training (e.g., teachers, doctors, psychotherapists), with *n* = 199 female, *n* = 101 male, and *n* = 2 non-binary participants. The average age was 43 years (*SD* = 14.7; ranging from 18 to 84 years). Among the sample, *n* = 133 (44%) indicated having experience with psychotherapeutic treatment (44%), *n* = 169 (56%) indicated not have made experiences with psychotherapeutic treatment so far. For additional sociodemographic characteristics please refer to Supplement S1.

### Measures

In the following section we describe the used assessment instruments and report their validity and reliability from the original questionnaires or translated German versions, wherever available.

#### AI aspects

The AI aspects ‘digital competence and AI awareness’ and ‘ PU of integrating AI into psychosocial care’ were assessed with eight items, formulated by McLennan et al. [64] for medical students. The original items were adapted to the general population and introduced as follows: “*The following is about your assessment of the use of Artificial Intelligence in the context of psychotherapy and mental health. Every day, we use so-called Artificial Intelligence, or AI for short. Most of the time without even realizing it. When we search for videos on YouTube, music on Spotify, or information on Google, or when we use Siri and Alexa, AI always plays an important role. Like humans, AI systems are capable of making decisions based on information. Similarly to us, AI systems can learn over time. For this, the AI needs as much information as possible. The more information an AI receives, the better it can solve the tasks and problems assigned to it and make decisions. Please read the following statements and indicate on the scale the extent to which you agree or disagree with the statements. There are no right or wrong answers.*” All items were rated on a 5-point Likert scale.

As the items were translated into German and adapted compared to the original version, we investigated the factor structure of the AI items by conducting an item analysis, difficulty analysis, and discrimination analysis as well as an exploratory factor analysis (EFA). Details on the results of the validation are presented in the Supplement S2, the German items are depicted in Supplement S4.

The first factor labelled"AI awareness and digital skills"comprises items 1 and 2 (1 = “I totally disagree” to 5 = “I fully agree”) and assesses digital skills and the subjective level of information on AI and mental health. This factor had low reliability with a Cronbach’s Alpha score of 0.511. The second factor, called"PU of integrating AI into psychosocial care"includes items 3 (1 = “I totally disagree” to 5 = “I fully agree”), 4 to 7 (1 = “not useful at all” to 5 = ”completely useful”) and 8 (1 = “not at all” to 5 = “very”) and covers the usefulness of 1) AI for mental health in general as well as for oneself, 2) AI programs such as ChatGPT for self-help, and 3) AI for mental health professionals in diagnosis, choice of treatment and the treatment itself. This factor had good reliability with a Cronbach’s Alpha of 0.842. For both sub-facets we calculated mean scores with higher scores indicating higher digital competence/AI awareness and PU, respectively.

Convergent validity was shown by significant correlation of Factor 1 with a scale on skills in the application of information technology for health (eHEALS [65, 66]) and low correlations with a scale on self-efficacy (ASKU [67]). For Factor 2 a low negative correlation with stigmatization (BMI [68, 69]) was observed. Detailed correlation coefficients are displayed in the online supplemental material accompanying this article (Supplement S2). ***Mental health app usage*** We developed a questionnaire on usage of mental health apps for this study. Participants were asked to indicate whether they use mental health-related apps (from never [0] to always [4]) and how many apps of this they have installed [0 to 15] on their smartphone. Subsequently, only those who indicated to have at least one app installed were asked on the frequency of their app usage (from never [0] to always [4]), as well as the apps’ content, referring to the following seven categories: Relaxation, meditation, and mindfulness exercises; Stress management; Daily structure and time management help; Mental health discussions (online platforms/forums); Sleep support; Digital diary; Support for mental disorder treatment (e.g., depression, anxiety). Usage was evaluated as a sum resulting in a maximum of 28 points with higher scores indicating a higher frequency of usage. Those who had no app installed were considered to have no usage.

#### Personality traits

The ***Big Five Inventory 10*** (BFI-10) by Rammstedt et al. [70] is a screening tool, which has been developed on the basis of the BFI, a 44-item questionnaire from John et al. [71] on the five basic dimensions of personality. It assesses each dimension with two items, using a 5-point Likert scale (1 = “does not apply at all"to 5 ="applies completely"): openness (item 5 and 10), consciousness (item 3 and 8), agreeableness (item 2 and 7), extraversion (item 1 and 6), and neuroticism (item 4 and 9). Retest reliability of the German version of the BFI-10 over 6 weeks was *r*_*TT*_ = 0.58—0.84; the internal consistencies as indicated by Cronbach’s Alpha for the scales in our sample are: openness α = 0.53, consciousness α = 0.50, agreeableness α = 0.30, extraversion α = 0.75 and neuroticism α = 0.57. It should be noted that the individual facets consist of only two items, which explains low consistencies [72]. By an EFA analysis and correlation with the sub facets of the NEO-PI-R [73] a good validity was shown for the BFI-10. The German version of the BFI-10 showed substantial convergent and discriminant validity, with some exceptions for agreeableness and openness. When calculating mean scores for each of the five personality dimensions, items 1, 3, 4, 5, and 7 of the BFI-10 were recoded; higher subscale scores indicate a higher expression of the respective personality trait [70, 74].

The ***Revised Life-Orientation-Test*** (LOT-R) by Scheier et al. [75] is a screening tool for assessing dispositional optimism. The LOT-R was translated into German first in the old version (LOT) by Wieland-Eckelmann & Carver (1990) [76]. The LOT was adapted to LOT-R and validated by Glaesmer et al. [77]. It consists of 10 items rated on a 5-point Likert scale (0 ="I disagree a lot"to 4 ="I agree a lot"), with 3 items being inverted (Items 3, 7 and 9). Retest reliability of 21–49 days was *r*_*TT*_ = 0.59 for optimism, *r*_*TT*_ = 0.65 for pessimism and *r*_*TT*_ = 0.75 for the full-scale. Validity wasn’t reported. In the present sample Cronbach’s Alpha is α = 0.786 for the pessimism scale and α = 0.795 for the optimism scale. For the LOT-R we computed a sum score for each of the two subscales using three items each (optimism: Items 1, 4 and 10; pessimism: Items 3, 7 and 9), and an overall optimism score using all items (including Items 3,7 and 9 as invers items) with higher scores indicating a higher optimism/pessimism for the subscales and only optimism for the total score. The items 2, 5, 6 und 8 are filler items and are not included in the calculation of the scores [77].

#### Mental health status

The ***General Health Questionnaire-12***(GHQ-12) by Goldberg et al. [78] is a short version of the originally developed 60-item GHQ [79] and is used as a screening tool for non-psychotic psychiatric disorders. In this study the German version of the GHQ-12 by Linden et al. [80] was used and validated among others by Schmitz et al. [81] and Romppel et al. [82]. All items are rated on a 4-point-likert scale (0 = “not at all” to 3 = “much more than usual”) and subjects are instructed to indicate the severity of psychological symptoms relative to the person’s normal situation during the last weeks. Higher scores indicate more psychological distress [82]. Internal consistency is α = 0.91 and the GHQ-12 showed good validity in receiver operating characteristics (ROC) analysis (*AUC* = 0.76, *SD* = 0.026) and by correlating the sum score (*r* = 0.37–0.73) with those of the sub facets of SCL-90-R [83]. For this sample the scale shows an internal consistency of α = 0.874. For calculating GHQ-12 scores, different scoring methods are possible. In this study the Likert scoring (0–1–2–3) resulting in a range from 0 to 36 was used for computing the sum score of GHQ-12 with higher scores indicating a greater likelihood of the presence of psychiatric disorders [81, 82].

The ***Kessler Psychological Distress Scale***(K10) by Kessler et al. [84] is a screening tool for assessing psychological distress. The German version of the K10 was translated and validated by Giesinger et al. [85]. It consists of 10 items rated on a 5-point-likert scale (1 ="none of the time"to 4 ="all the time"). Subjects are instructed to indicate their emotional state during the last four weeks. Validation in two samples demonstrated a satisfactory level of internal consistency (α = 0.80—0.90). Substantial convergent validity was demonstrated through correlations with subscale for trait anxiety of the STAI [86, 87] (*r* = 0.68) and the global severity index of the BSI [88, 89] (*r* = 0.71). For this sample the scale shows an internal consistency of α = 0.918. For the K10 we calculated a sum score for all items with higher scores indicating higher psychological distress [85].

The ***Perceived Stress Scale***(PSS 2&2) by Schäfer et al. [90] was developed by Cohen et al. [91] and originally included 14 items, assessing subjectively perceived stress. Recently, Schäfer et al. [90] developed a German short version of the scale which consists of 4 items rated on a 5-point-likert scale (1 = “never” to 5 = “very often”). In line with the original questionnaire and German versions of the PSS-10 [92, 93], factor analyses revealed two sub-facets: perceived helplessness and perceived self-efficacy. Internal consistency was ω = 0.85 for helplessness and ω = 0.84 for self-efficacy. Validity was shown through factor analysis and network modelling. For this sample the scale shows an internal consistency of α = 0.61 for helplessness and α = 0.78 for self-efficacy. For the PSS 2&2 we calculated a sum score for each sub-facet with higher scores indicating higher level of helplessness and self-efficacy, respectively [90].

The ***Brief Resilience Scale***(BRS) by Smith et al. [94] is a screening tool for assessing the individual ability to recover from stress. The German version of the BRS was translated and validated by Chmitorz et al. [95]. It consists of 6 items with items 2, 4, and 6 being reversed. All items are rated on a 5-point-likert scale (1 = “strongly disagree” to 5 = “strongly agree”). Internal consistency was α = 0.85. Validity was shown by positive correlations with well-being, social support, optimism, and the coping strategies active coping, positive reframing, acceptance, and humor as well as negative correlations with somatic symptoms, anxiety and insomnia, social dysfunction, depression, and the coping strategies religion, denial, venting, substance use, and self-blame. For this sample the scale shows an internal consistency of α = 0.82. For the BRS a sum score with all items is calculated, with higher scores indicating higher levels of resilience [95].

#### Mental health knowledge, attitude towards mental health, and help-seeking

The **Mental Health Literacy Scale** (MHLS) by O’Connor & Casey [96] is a questionnaire assessing MHL. The German version [97] was reached by translation of the English original from O’Connor & Casey [96]. Based on item and factor analyses, the German version consists of 31 items (instead of originally 35 items) with 9 inverse items (item 21–28). 11 Items are rated on 4-point-likert-scale from ranging from 0 (very unlikely) to 3 (very likely) and 20 items are rated on a 5-point-likert-scale ranging from 0 (do not agree at all) to 4 (fully agree) and from 0 (definitely not ready) to 4 (definitely ready). Our validation study for the German version revealed four factors (social distance, stigmatization, information seeking and knowledge) representing the following two facets of MHL ([1] knowledge and [2] attitude and skills) and a total sum score for MHL. The four factors demonstrated satisfactory to excellent internal consistencies (α = 0.75—0.91). The sub-facet ‘knowledge’ had an internal consistency of α = 0.727 and the sub-facet ‘attitude and skills’ an internal consistency of α = 0.864. We also showed substantial convergent validity of the MHLS, indicated by significant correlations with the Social Distance Scale [98–101], the GHSQ [102, 103] and MHLq-YA [104] (*r* = 0.330, 0.284 and 0.669) as well as divergent validity reflected by low correlations with the K10 [84] and both sub-facets of LOT-R [75] (*r* = 0.014, 0.047 and −0.118), whereby the only significant correlation was those with LOT-R pessimism. The use of the MHLS in this study included only the second level sub-facets of the MHLS. Higher scores indicate more MHL in the sense of more knowledge respectively lower stigmatization, higher ability to seek information and lower social distance.

The ***Inventory of Attitudes Towards Seeking Mental Health Services*** (IASMHS) by Mackenzie et al. [105] is a questionnaire for assessing attitudes toward seeking professional help. It is a modified version of Fischer and Turner’s [106] Attitudes Towards Seeking Professional Psychological Help Scale (ATSMHS). The German version of this questionnaire was translated and validated by Kessler et al. [107]. It consists of 24 items rated on a 5-point Likert scale (0 = “disagree” to 4 = “agree”) with 15 inverse items (item 1, 3, 4, 6, 7, 9, 11, 12, 14, 16–18, 20, 21, 24). Validation studies have demonstrated a satisfactory level of internal consistency across all items (α = 0.79) and for the three subscales (psychological openness, help seeking propensity, indifference to stigma; α = 0.67—0.84). For this sample the scale shows an internal consistency of α = 0.537 for the full-scale, α = 0.704 for psychological openness, α = 0.784 for help seeking propensity and α = 0.647 for indifference to stigma. For the IASMHS sum scores for each of the three subscales are calculated with higher scores indicating higher level on the sub-facet (psychological openness: items 1, 4, 7, 9, 12, 14, 18, 21; help seeking propensity: items 2, 5, 8, 10, 13, 15, 19, 22, indifference to stigma: 3, 6, 11, 16, 17, 20, 23, 24) and for the full-scale [107].

The ***General Help Seeking Questionnaire*** (GHSQ) by Wilson et al. [103] and Hammer & Spiker [102] is a questionnaire assessing help seeking intentions for personal-emotional problems. It was translated to German and adapted for this study from the versions of Wilson et al. [103] and Hammer & Spiker [102]. It originally consists of 10 items, 4 for an informal source, 4 for a formal source, one item for “no one” and one item for “another source” [102, 103]. We adapted the German version of the GHSQ to include for example work colleagues as informal source and to differentiate more detailed between different types of professional help, resulting in a total of 15 items (the respective Cronbach’s alpha values for the different help-seeking dimensions are given in parentheses): 5 items for an informal source (partner, friends, parents, other relatives or family members, work colleagues; α = 0.553), 4 items for a formal, but nonprofessional source (non-medical practitioners for psychotherapy, online counselling/internet services, telephone counselling [e.g. telephone counselling], religious/spiritual counsellors [e.g. pastoral care]; α = 0.434), 4 items for a professional source (psychotherapist, psychiatrist, psychological/psychosocial/psychotherapeutic counselling centre, general practitioner; α = 0.675), one item for “no one” and one item for “another source”. All items are rated on a 4-point-likert scale (1 = “extremely unlikely” to 6 = “extremely likely”). To date, there is no validation for the German version. For the present study, we computed a mean score for each of the help-seeking dimensions: informal, formal but non-professional, and professional help seeking. Higher scores indicated higher help seeking regarding the respective facet.

#### Data preparation and analysis

Data preparation included the recoding of inverted items and calculation of subscale and total scores according to the specified guidelines and as outlined above. Subscale and total scores were only calculated in case of no missing values on that questionnaire.

For data analysis, IBM SPSS Statistics 23.0.0 for Windows (IBM Corp., 2020) was utilized. The significance level for all tests was set at *p* < 0.05.

For the first and second research questions we used stepwise regression analyses with PU of integrating AI into psychosocial care and actual use of related apps as dependent variables, respectively. The regression analyses offer the possibility to simultaneously control variables (by taking into account the variance explained by a variable, it is ensured that the reported effects of other included independent variables are not confounded) and still report on their influence (the coefficient reflects the amount of change in the dependent variable due to an one-unit increase, whereas holding all other variables constant) on the dependent variable. To identify the unique contributions of the variables to the explained variance of the dependent variable and to account for effects of the sociodemographic and other basic variables, we progressively introduced predictors across three blocks. Categorical and non-dichotomous variables (such as gender and group variables) were dummy coded prior to analysis. In Block 1 we entered age, gender, psychotherapeutic experience, AI awareness and digital skills as well as a group variable representing the general population, pastoral caregivers, and individuals with psychological-pedagogical basic training using the inclusion method to account for and control the effects of these variables. In Block 2, personality factors were introduced in a stepwise manner (inclusion criteria: probability of F-value for inclusion < = 0.050, probability of F-value for exclusion > = 0.100), encompassing measures of neuroticism, extraversion, openness, conscientiousness, agreeableness assessed by the BFI-10 [74], along with optimism, and pessimism assessed by LOT-R [75, 77]. Finally, Block 3 included variables related to mental health, stress and resilience [(GHQ-12 [78, 80]; K10 [84, 85]; PSS-2 × 2 [90]; BRS [94, 95]], mental health knowledge [(MHLS [96, 97]), attitudes towards mental health (IASMHS [105, 107]), and help seeking behaviour (GHSQ [102, 103]), in a stepwise manner (inclusion criteria: probability of F-value for inclusion < = 0.050, probability of F-value for exclusion > = 0.100).

We calculated Power analysis using G*Power (version 3.1.9.6) which showed excellent power (> 99%) for both regression analyses. Furthermore, we checked assumptions for both regression analyses. For the first regression analysis (1) the assumption of a linear relationship between variables was met and (2) we observed no autocorrelation in the residuals as indicted by the Durbin-Watson test yielding a value of 2.192; (3) residuals were found to be normally distributed; 4) no multicollinearity was present and 5) variances were homogeneous. Finally, no outliers were detected in the data. For the second regression analysis, the assumption check showed (1) a linear relationship between variables; (2) no autocorrelation in the residuals as indicated by the Durbin-Watson test yielding a value of 1.959; (3) no multicollinearity and (4) homogeneity of variance. There were no outliers detected in the data, but the residuals were found to be not normally distributed. However, the multiple regression analysis can be considered sufficiently robust regarding violations of the assumptions.

Finally, the moderation analyses was conducted using PROCESS Procedure for SPSS Version 4 [108]. We performed two moderation analyses with two indicators of current mental health burden as moderator: one with the moderator mental health symptoms during the last weeks, as measured with the GHQ-12, and a second with the moderator current psychological distress, as measured with the K-10. For both moderation analyses PU of integrating AI into psychosocial care was used as predictor variable and actual mental health app usage as outcome. Working area (population, pastoral caregiver and persons with pedagogical/psychological training) and digital competence and AI awareness were included as covariates. The assumptions for moderation analysis (linearity and independency of errors) were fulfilled (normal distribution) or the violation was prevented by robust procedures (normal distribution of residuals and homoscedasticity). The relationship between the variables appeared approximately linear upon visual inspection of the scatterplot with LOESS smoothing, with deviations observed in the outer ranges. Since the independence of observations is given, the cases cannot be related to each other via the outcome because they do not share any characteristics or correlations. The independence of the errors is given accordingly. Post-hoc Power analysis using G*Power (version 3.1.9.6) showed excellent power (> 99%) for moderation analysis with the whole sample.

## Results

### Perceived usefulness of integrating AI into psychosocial care (RQ 1)

Within the first stepwise regression analysis (*n* = 269) seven models were computed to identify significant predictors of PU of integrating AI into psychosocial care. The final model (*F*(13, 255) = 6.861, *p* < 0.001) revealed a total variance explanation of *R*^*2*^ = 0.259 (adjusted *R*^*2*^ = 0.221). From block 1 (entering method) working field (population, pastoral caregiver or psychological/pedagogical trained), treatment experience, digital competence and AI awareness were significant predictors of PU of integrating AI into psychosocial care. Among the significant predictors working field and treatment experience were negatively related, whereas digital competence and AI awareness was positively related to PU. From block 2 (stepwise method), the personality factors agreeableness, openness, pessimism and conscientiousness were included in the model as significant predictors with agreeableness being positively and openness, pessimism and conscientiousness being negatively related to PU. Finally, from the third block, the MHLS sub facet ‘attitude and skills’, for which higher scores indicate higher MHL (in the sense of lower social distance, stigmatization, and higher ability of information seeking), and the IASMHS sub facet ‘psychological openness’ were included in the model as significant predictors. Whereas the MHLS sub facet ‘attitude and skills’ were positively related to PU, psychological openness was negatively related to PU of integrating AI into psychosocial care. Detailed statistical indices for significant predictors are displayed in Table 1.

**Table 1:** Stepwise hierarchical regression analysis predicting the PU of integrating AI into psychosocial care (*N* = 269) with statistics for the significant models and for the variables included in the respective models. Statistical indices of the variables excluded from the respective regression models are depicted in the Online Supplement File S3

	β	*T*	*p*	*R*^*2*^	*R*^*2*^* Adj*	*F (Model)*	*p (Model)*
**Model 1**							
B1: Dummy pastoral caregiver	−0.15	−2.29	.023*				
B1: Dummy psychol./pedag. training	−0.14	−2.24	.026*				
* B1: Age*^#^	*0.07*	*1.03*	*.306*				
* B1: Dummy women*	*−0.04*	*−0.12*	*.907*				
* B1: Dummy men*	*−0.15*	*−0.45*	*.651*				
B1: Treatment experience	−0.12	−2.16	.032*				
B1: Digital competence/AI awareness	0.35	5.72	<.001***				
**Model Statistics**				.16	.14	6.98	<.001***
**Model 2**							
B1: Dummy pastoral caregiver	−0.17	−2.56	.011*				
B1: Dummy psychol./pedag. training	−0.15	−2.42	.016*				
* B1: Age*	0.06	*0.91*	*.365*				
* B1: Dummy women*	−0.11	*−0.36*	*.723*				
* B1: Dummy men*	−0.22	*−0.69*	*.488*				
B1: Treatment experience	−0.12	−2.05	.042*				
B1: Digital competence/AI awareness	0.33	5.50	<.001***				
B2: Agreeableness (BFI-10)	0.16	2.72	.007**				
**Model Statistics**				.18	.16	7.18	<.001***
**Model 3**							
B1: Dummy pastoral caregiver	−0.17	−2.57	.011*				
B1: Dummy psychol./pedag. training	−0.16	−2.47	.014*				
* B1: Age*	*0.09*	*1.29*	*.198*				
* B1: Dummy women*	*−0.14*	*−0.43*	*.666*				
* B1: Dummy men*	*−0.26*	*−0.80*	*.422*				
* B1: Treatment experience*	−0.11	−1.94	*.054*				
B1: Digital competence/AI awareness	0.34	5.67	<.001***				
B2: Agreeableness (BFI-10)	0.16	2.81	.005**				
B2: Openness (BFI-10)	−0.12	−2.07	.040*				
**Model Statistics**				.19	.17	6.94	<.001***
**Model 4**							
B1: Dummy pastoral caregiver	−0.19	−2.90	.004**				
B1: Dummy psychol./pedag. training	−0.16	−2.53	.012*				
* B1: Age*	*0.08*	*1.29*	*.199*				
* B1: Dummy women*	*−0.18*	*−0.58*	*.560*				
* B1: Dummy men*	*−0.32*	*−1.00*	*.321*				
* B1: Treatment experience*	*−0.10*	*−1.85*	.*066*				
B1: Digital competence/AI awareness	0.35	5.78	<.001***				
B2: Agreeableness (BFI-10)	0.13	2.16	.032*				
B2: Openness (BFI-10)	−0.14	−2.39	.017*				
B2: Pessimism (LOT-R)	−0.13	−2.06	.040*				
**Model Statistics**				.21	.18	6.75	<.001***
**Model 5**							
B1: Dummy pastoral caregiver	−0.19	−2.93	.004**				
B1: Dummy psychol./pedag. training	−0.16	−2.53	.012*				
* B1: Age*	*0.10*	*1.44*	*.151*				
* B1: Dummy women*	*−0.22*	*−0.71*	*.476*				
* B1: Dummy men*	*−0.37*	*−1.17*	*.245*				
* B1: Treatment experience*	*−0.11*	*−1.90*	*.059*				
B1: Digital competence/AI awareness	0.36	6.08	<.001***				
B2: Agreeableness (BFI-10)	0.13	2.16	.032*				
B2: Openness (BFI-10)	−0.13	−2.27	.024*				
B2: Pessimism (LOT-R)	−0.16	−2.52	.012*				
B2: Conscientiousness (BFI-10)	−0.13	−2.27	.024*				
**Model Statistics**				.22	.19	6.70	<.001***
**Model 6**							
B1: Dummy pastoral caregiver	−0.20	−2.99	.003**				
B1: Dummy psychol./pedag. training	−0.17	−2.81	.005**				
* B1: Age*	*0.12*	*1.79*	*.075*				
* B1: Dummy women*	*−0.15*	*−0.49*	*.624*				
* B1: Dummy men*	*−0.29*	*−0.92*	*.360*				
B1: Treatment experience	−0.15	−2.57	.011*				
B1: Digital competence/AI awareness	0.37	6.19	<.001***				
B2: Agreeableness (BFI-10)	0.12	2.05	.041*				
B2: Openness (BFI-10)	−0.14	−2.40	.017*				
B2: Pessimism (LOT-R)	−0.13	−2.07	.040*				
B2: Conscientiousness (BFI-10)	−0.15	−2.57	.011*				
B3: Attitudes and Skills (MHLS)	0.17	2.85	.005**				
**Model Statistics**				.25	.21	6.99	<.001***
**Model 7**							
B1: Dummy pastoral caregiver	−0.18	−2.70	.007**				
B1: Dummy psychol./pedag. training	−0.17	−2.68	.008**				
* B1: Age*	*0.13*	*1.93*	*.055*				
* B1: Dummy women*	*−0.19*	*−0.62*	*.538*				
* B1: Dummy men*	*−0.34*	*−1.10*	*.271*				
B1: Treatment experience	−0.14	−2.42	.016*				
B1: Digital competence/AI awareness	0.36	6.04	<.001***				
B2: Agreeableness (BFI-10)	0.12	2.12	.035*				
B2: Openness (BFI-10)	−0.13	−2.30	.023*				
B2: Pessimism (LOT-R)	−0.17	−2.57	.011*				
B2: Conscientiousness (BFI-10)	−0.15	−2.59	.010*				
B3: Attitudes and Skills (MHLS)	0.24	3.52	.001**				
B3: Psychological Openness (IASMHS)	−0.15	−2.07	.040*				
**Model Statistics**				.26	.22	6.86	<.001***

Only significant predictors are displayed for the respective models (for non-significant predictors see Supplement S3). *p* <.050*; *p* <.010**; *p* <.001***; B1/2/3 = Block 1/2/3; Adj, adjusted; β, standardized beta coefficient; ^#^variables that were forced into the model in the first block but were not significant are shown in italic

### Usage of mental health apps (RQ 2)

For the second stepwise regression with the frequency of mental health app usage (*n* = 276) as dependent variable, a total of three models was computed to identify significant predictors. The final model (*F*(9,266) = 7.299, *p* < 0.001) showed a variance explanation of *R*^*2*^ = 0.198 (adjusted *R*^*2*^ = 0.171).

From block 1, digital competence and AI awareness was included in the model as significant positive predictor for the frequency of mental health apps usage. From block 2, no variable was included in the regression analysis based on the criteria (probability of F-value for inclusion < = 0.050, for exclusion > = 0.100). Finally, from block 3, psychological distress, and formal, but non-professional help seeking were included as significant predictors in the final model with both being positively related to the frequency of mental health app usage. Detailed statistical indices for significant predictors are displayed in Table 2.

**Table 2:** Stepwise hierarchical regression analysis predicting the frequency of mental health-related app usage (*N* = 276) with statistics for the significant models and for the variables included in the respective models. Statistical indices of the variables excluded from the respective regression models are depicted in the Online Supplement File S3

	β	*T*	*p*	*R*^*2*^	*R*^*2*^* Adj*	*F (Model)*	*p (Model)*
**Model 1**							
* B1: Dummy pastoral caregiver*^#^	*0.09*	*0.18*	*.855*				
B1: Dummy psychol./pedag. training	*0.41*	*0.66*	*.507*				
B1: Age	−0.04	−2.21	.028*				
* B1: Dummy women*	−3.26	−1.36	.190				
* B1: Dummy men*	−4.13	−1.65	.101				
B1: Treatment experience	1.21	2.87	.004**				
B1: Digital competence/AI awareness	0.74	2.72	.007**				
**Model Statistics**				.14	.11	6.04	<.001***
**Model 2**							
* B1: Dummy pastoral caregiver*	*0.32*	*0.67*	*.503*				
* B1: Dummy psychol./pedag. training*	*0.60*	*0.99*	*.324*				
* B1: Age*	−0.03	−1.51	.132				
* B1: Dummy women*	−3.21	−1.32	.188				
* B1: Dummy men*	−3.86	−1.57	.118				
* B1: Treatment experience*	*0.77*	*1.76*	*.080*				
B1: Digital competence/AI awareness	0.76	2.82	.005**				
B2: Psychological Distress (K-10)	0.11	3.34	.001**				
**Model Statistics**				.17	.15	6.88	<.001***
**Model 3**							
* B1: Dummy pastoral caregiver*	*−0.16*	*−0.31*	.*756*				
* B1: Dummy psychol./pedag. training*	*0.54*	*0.90*	*.371*				
* B1: Age*	−0.03	−1.86	.065				
* B1: Dummy women*	−3.02	−1.26	.209				
* B1: Dummy men*	−3.63	−1.50	.136				
* B1: Treatment experience*	*0.83*	*1.92*	*.056*				
B1: Digital competence/AI awareness	0.79	2.97	.003**				
B2: Psychological Distress (K-10)	0.11	3.44	.001**				
B2: Formal but non-professional help seeking (GHSQ)	1.06	3.00	.003**				
**Model Statistics**				.20	.17	7.30	<.001***

Only significant predictors are displayed for the respective models (for non-significant predictors see Supplement S3). *p* <.050*; *p* <.010**; *p* <.001***; B1/2/3 = Block 1/2/3; Adj, adjusted; β, standardized beta coefficient; ^#^variables that were forced into the model in the first block but were not significant are shown in italic

### Psychological Distress, AI Utility, and Mental Health Apps (RQ 3)

Two moderation analyses were run to determine whether the interaction between PU of integrating AI into psychosocial care and mental health problems and psychological distress, respectively, significantly predicts the usage of mental health apps when controlling for the working background (general population, pastoral care, pedagogical/psychological training) and digital competence and AI awareness as covariates.

For the moderator mental health problems (as measured with the GHQ-12), the overall model was significant *F*(5,275) = 3.22, *p* = 0.008, predicting 11.14% of the variance in mental health app usage. The GHQ-12 sum score did, however, not significantly moderate the relationship between PU of integrating AI into psychosocial care and the frequency of mental health app usage (*ΔR*^*2*^ = 1.89%, *F*(1, 275) = 2.77, *p* = 0.097). For statistical details on the main and interaction as well as covariate effects see Table 3.

**Table 3:** Results of the moderation analysis with PU of integrating AI into psychosocial care as predictor, frequency of mental health app usage as outcome and mental health problems as moderator

	*coefficient*	*SE (HC3)*	*t*	*p*	*LLCI*	*ULCI*
Perceived usefulness of integrating AI into psychosocial care	0.54	0.34	1.60	.110	−0.12	1.20
Mental health problems (GHQ-12)	0.15	0.06	2.40	.017*	0.03	0.27
Interaction effect	0.11	0.07	1.66	.100	−0.02	0.25
Group variable	0.27	0.36	0.75	.454	−0.44	0.97
Digital competence and AI awareness	0.76	0.30	2.56	.011*	0.17	1.34

*p* <.050*; *p* <.010**; *p* <.001***

For the moderator psychological distress (as measured with the K-10), the overall model was significant, *F*(5,282) = 5.40, *p* < 0.001, predicting 20.78% of the variance. Results indicate that psychological distress moderates the effect between PU of integrating AI into psychosocial care and the frequency of mental health app usage (*ΔR*^*2*^ = 4.36%, *F*(1, 282) = 10.76, *p* = 0.0012, 95% *CI*[0.06, 0.23]). For statistical details on the main and interaction as well as covariate effects see Table 4.

**Table 4:** Results of the moderation analysis with PU of integrating AI into psychosocial care as predictor, frequency of mental health app usage as outcome and psychological distress as moderator

	*coefficient*	*SE (HC3)*	*t*	*p*	*LLCI*	*ULCI*
Perceived usefulness of integrating AI into psychosocial care	0.88	0.31	2.85	.005**	0.27	1.50
Psychological Distress (K10)	0.15	0.04	3.64	<.001***	0.07	0.24
Interaction effect	0.14	0.04	3.28	.001**	0.06	0.23
Group variable	0.64	0.35	1.84	.067	−0.04	1.33
Digital competence and AI awareness	0.64	0.27	2.31	.021*	0.10	1.18

*p* <.050*; *p* <.010**; *p* <.001***

To further examine the significant interaction the conditional regression coefficients were estimated (for details see Table 5). For individuals with below-average psychological distress (one standard deviation below the mean), the PU of integrating AI into psychosocial care was not a significant predictor of the frequency of mental health app usage (*p* = 0.739). For those with average and above-average psychological distress (one standard deviation above the mean), PU of integrating AI into psychosocial care was a significant predictor of the frequency of mental health app usage (both *p* < 0.01).

**Table 5:** Conditional effects of the focal predictor at values of the moderator

Psychological Distress (K10)	*Effect*	*SE (HC3)*	*t*	*p*	*LLCI*	*ULCI*
−7,16	−0.13	0.38	−0.33	.739	−0.87	0.62
0.00	0.88	0.31	2.85	.005**	0.27	1.50
7.16	1.89	0.49	3.87	<.001***	0.93	2.86

*p* <.050*; *p* <.010**;* p* <.001***; Outcome Variable: frequency of mental health app usage. K10 [84, 85]

## Discussion

The aim of this study was to investigate the influence of individual factors related to personality and mental health (big five personality dimensions, symptoms of mental impairments, psychological distress, resilience, mental health literacy) on the PU of integrating AI into mental health care and the actual use of mental health apps based on the technology acceptance model, while accounting for other factors previously suggested to influence PU and actual use (e.g., sociodemographic variables, digital abilities) as covariates. For PU of integrating AI into psychosocial care our analysis revealed that working area, more treatment experience, higher levels of openness, pessimism, conscientiousness and psychological openness (towards help-seeking) negatively predicted the PU, whereas higher digital capabilities, more agreeableness, and higher scores on the attitude and skill facet of MHL (in the sense of lower stigma and social distance and better ability of information seeking) positively predicted PU. With respect to actual usage, we observed that the use of mental health apps significantly increased with digital capabilities, psychological distress, and help seeking in formal but non-professional settings, whereas—in contrast to the PU—personality traits had no influence on the frequency of app-usage. The relationship between PU of integrating AI into psychosocial care and the actual use of mental health app – irrespective of AI integration—was significantly moderated by psychological distress. For those with average and above-average psychological distress, PU of integrating AI into psychosocial care was a significant predictor of the frequency of mental health app usage.

In contrast to most previous studies [29, 31–33], but in line with some others [30, 34] we found no significant influence of age on PU of AI usage in psychosocial care or the usage of mental health apps. Further, we did not find significant effects of gender neither on the PU nor the usage of mental health apps, which is in accordance to some previous results, as, so far, other empirical findings on the influence of gender on PU or app usage were also mixed [3, 29, 30]. The lack of a significant influence of age and gender on PU and App usage in our study might – at least in part—be explained by overlapping, variance-revealing effects of other variables that had a significant influence on the PU and actual use of mental health apps. First, our sample was relatively highly educated which appears to be associated with higher AI acceptance in general [30] and possibly also with digital capabilities and AI awareness, which in turn was a highly significant predictor of PU and for app usage in our study as well as in previous studies [3, 29, 34]. Second, treatment experience was an additional significant predictor of PU (and tended to be a predictor of app use), which may have overshadowed other effects. The fact that the age effects are in the trend range of significance and thus certainly show an influence that is not significant in the overall model could also speak in favour of our explanation of superimposed effects. In accordance with existing evidence, the professional field was a significant predictor of the PU of AI in psychosocial care but not for app use. Persons working in areas associated with psychosocial care had less PU of integrating AI into psychosocial care. In attempting to explain this effect, numerous other studies have suggested that employees in a psychosocial sector worry about being replaced by AI in this area and thereby losing their job [e.g. 3, 110]. Furthermore, people working in the mental health sector may have a greater sensitivity to the critical factors of AI use in relation to health-related data and data security issues, which could ultimately lead to a lower PU in relation to AI [6, 110].

Mostly consistent with existing evidence and the TAM2, our study revealed a significant influence of various personality traits on PU of integrating AI into psychosocial care [19]. In line with previous studies about communicative AI [30] and with a sample from the Amazon MTurk participant pool [30, 36], agreeableness was a positive predictor of PU in the present study. In contrast, our result that conscientiousness and pessimism both negatively predicted PU, is in contrast to previous research which found no significant influence of conscientiousness on attitudes towards AI [30, 36]. Furthermore, Salar und Hamutoglu [111] observed no influence of the personality traits extraversion, agreeableness and conscientiousness, but a significant influence of nervousness on PU when investigating the acceptance of Cloud Computing Systems. Finally, the personality trait openness was negatively associated with PU in the present study, while other studies showed a positive relationship between the openness to experience and AI technologies [30]. The type of questions in different questionnaires may account for contradictory results as the two items covering the openness factor within the BFI-10 primarily ask about artistic interest and creativity, whereas other questionnaires (e.g. ten item personality measure [112]) focus on openness to experiences (e.g., “I am someone who is curious about many different things”). Furthermore, in a complex regression model, openness lost its significant contribution, when AI anxiety was introduced in the model [34]. Overall, results on the influence of personality on PU and attitudes towards AI are heterogeneous [113] and additional research in large samples and measures of various psychological constructs is needed to disentangle the role of personality factors within a larger network of predictors for PU.

Finally, our results on the influence of stigmatising attitudes on PU were not in line with previous research. Whereas in our study PU of integrating AI into psychosocial care increased with higher MHL, that means with less stigmatization and social distancing as well as a better ability to search for information, other studies suggested that individuals with stronger stigmatizing attitudes might have the impression of greater privacy and a reduced fear of judgement when using non-personal mental health apps [3, 6, 31]. In our study, higher psychological openness was negatively associated with the PU of AI in mental health care, which may be explained by the fact that psychologically more open individuals prefer personal contact to digital or AI applications because they have less fear of stigmatisation in these personal contacts or because they already have more experience in the area of mental health. In university students, it has been, for example shown, that contact to mentally ill people in the immediate vicinity and experience in the mental health field lead to higher psychological openness [114].

Interestingly, the predictors for the two outcomes, i.e. PU and actual app usage, did not overlap at all, with the exception of digital competences and AI awareness, which predicted both PU and actual app usage. Neither socio-demographic variables nor personality traits had a significant influence on actual app usage, which can be viewed in line with the TAM2, proposing that external factors indirectly influence app usage via PU [19, 24]. Longitudinal study designs and subsequent data analyses with structure equation modelling would help to identify direct and indirect pathways between individual and environmental predictors of PU and actual app usage as well as between these variables. Our results that app usage is influenced by psychological distress and a higher intention to seek formal, non-professional help (e.g., online counselling/internet services, telephone counselling, religious/spiritual counsellors) is in line with other studies showing that app usage is primarily influenced by mental health concerns, help seeking, and the social environment [32, 40, 41]. Further empirical evidence showing a significant influence of perceived stress on the acceptance of stress coping apps suggests that utilizing mental health apps is predicted by the actual need for support [115, 116]. However, to further delineate the relationship between different state and trait variables in predicting the usage of mental health apps, future studies using an ecological momentary assessment design for the collection of data on stress exposure, mental health and user data of mental health apps are needed.

Psychological distress was also a significant moderator of the relationship between PU of integrating AI in psychosocial care and the actual frequency of app usage—irrespective of AI integration and even when controlling for technological openness—as starting and end point of the TAM [32, 39–41]. In contrast, mental health as measured by the GHQ-12 was no significant moderator, suggesting that – in the case of mental health apps—the stress level rather than current mental health problems drives an individual to transform an attitude, here PU, into action, here app usage. High mental stress might lead to a higher frequency of use of AI-supported apps for mental health issues due to problems in the German care system with regard to timely access to psychotherapeutic support [117]. In addition, corresponding apps offer low-threshold access to self-help even in the case of emerging stress that is not yet pathological or chronified. In this vein, it has to be considered that besides psychological distress the intention to seek formal, non-professional help predicted mental health app use, i.e., a support service that is one example for formal, non-professional help. Future studies might differentiate different types of support services for mental health problems as endpoint and investigate the individual determinants as well as circumstances under which they are sought out. In contrast to our finding, Borghouts et al. [40] found no direct effect of perceived stress on mental health app usage, yet in a mediation analysis the study revealed that the perceived need to seek help significantly mediated the relationship between perceived stress and mental health app use. Altogether these results suggest that the individual stress level is relevant for accepting AI in the health care sector and, ultimately, for using mental health related apps.

In summary, aspects of mental health literacy (e.g. stigmatising attitude, psychological openness) seem to particularly influence the PU of AI involvement in mental health care, while aspects of mental health (e.g. psychological distress, help seeking intention) seem to particularly influence the use of mental health apps (without a specific AI focus). The correlation between these two aspects was only moderated when psychological distress was average and above-average, which provides initial indications that future AI-based applications may have to be specifically tailored to the needs of different target groups and that it could also be important to make the applications more attractive as preventive or low-threshold offers for less stressed groups.

## Limitations

The results of the present study have to be interpreted in the light of some limitations. First, our sample is not representative neither for the German general population nor the included subgroups. Due to the online nature of the survey, the recruitment strategy can have led to self-selection among participants and there seems to be an overrepresentation of participants with good digital knowledge highly educated, young or middle-aged. Participation was voluntary, driven by self-motivation and financial incentives, potentially impacting the generalizability of results based on this sample. Both the lack of representativeness in the composition of the sample and the likely presence of self-selection constrains the external validity of the findings. Second, when using self-reporting in surveys, particularly for retrospective statements regarding the frequency and intensity of past or future experiences and behaviours like for example the app usage, systematic biases and inaccuracies may arise due to the retrieval of memory information and the influence of social phenomena, such as social desirability or response tendencies. In future studies it would therefore be interesting to apply an ecological momentary assessment approach and collect user data of mobile devices (e.g. smartphones) in order to avoid such biases. Another limitation is the unequal group size of the different occupational groups. The group size of the psychologically and pedagogically trained persons was significantly smaller in relation to the other two groups, which is why a subgroup analysis might have shown an existing difference between the groups to be non-significant. For this reason, subgroup analyses were not performed. Finally, another limitation is that we only considered the influence of external variables on PU and actual usage as important parts of the TAM/TAM2, whereas we did not assess the attitude towards and the intention to use mental health apps, which would have allowed to evaluate the complete TAM/TAM2. However, as data assessment was carried out in the context of a larger study, we had time constraints and opted for a more detailed and fine-grained assessment of external and control variables. A comprehensive explanation of the relationships, mediating and moderating effects of the variables contained in TAM/TAM2 is therefore not possible based on our selection of examined variables. When replicating and extending our results, it is therefore advisable to include not only the start and end points of the TAM (PU and current use) but also attitudes towards use and the intention to use. Due to ethical and data protection difficulties in the integration of AI in mental health apps in Germany, usage was surveyed across the board as part of this study and did not only include the use of mental health apps with AI integration. Accordingly, only initial conclusions can be derived from the connection between PU and use in this study.

## Conclusion

By investigating different predictors of PU of integrating AI into psychosocial care and actual mental health app use, the present study revealed that whereas the PU is related to differences in socio-demographic data, competences, and personality traits, the frequency of actual app use is only related to psychological distress and the intention to use formal, non-professional help. Psychological distress, moreover, was a significant moderator of the relationship between PU and actual usage of mental health apps in general. These results suggest that different target variables (such as personality traits, skills, age, gender, mental health status) need to be addressed to promote different aspects of AI in the healthcare sector, such as attitudes towards AI in healthcare on the one hand and the actual use of AI tools on the other. Results of the present study also add to existing theoretical knowledge as we provide evidence for the applicability of the TAM/TAM2 in the field of digital mental health applications. Yet, it appears that, with respect to digital mental health applications, a direct connection between PU and usage exists, which is not mediated by intention. As the intention itself was not assessed in the present study, further research has to investigate the relationship between the distinct aspects of technology acceptance models in more detail.

Our results suggest that the mental health status itself and factors related to mental health may be useful to consider in the design and promotion of mental health apps with the ultimate goal to improve their acceptability and usage. People with mental impairments face particular psychological challenges [118, 119], but they do not appear to find the existing applications sufficiently useful in contrast to more generally distressed individuals. Thus, tailoring digital mental health applications to the needs of particular subgroups of users, e.g. individuals with mental health conditions or with specific personality traits, could prove useful.

## Supplementary Information

Supplementary Material 1

Supplementary Material 2

Supplementary Material 3

Supplementary Material 4

## Data Availability

The datasets used for the analysis of the current paper are available from the corresponding author.

## Abbreviations

AI: Artificial intelligence
*BMI*: Beliefs toward Mental Illness Scale
BFI-10: Big Five Inventory 10
BRS: Brief Resilience Scale
BSI: Brief Symptom Inventory
DL: Deep learning
eHEALS: EHealth Literacy Scale
TAM: Extended Technology Acceptance Model
GHSQ: General Help Seeking Questionnaire
GHQ-12: General Health Questionnaire – 12
ASKU: General Self-Efficacy Short Scale
HITAM: Health Information Technology Acceptance Model
IASMHS: Inventory of Attitudes Towards Seeking Mental Health Services
JGU Mainz: Johannes Gutenberg-University Mainz
K10: Kessler Psychological Distress Scale
LIR: Leibniz-Institute for Resilience Research
LOT-R: Life-Orientation-Tests
ML: Machine learning
MHL: Mental Health Literacy
MHLS: Mental Health Literacy Scale
MHLq-YA: Mental Health Literacy Questionnaire in Young Adults
NEO-PI-R: NEO personality inventory
NLP: Natural language processing
PSS 2&2: Perceived Stress Scale
PU: Perceived usefulness
PEU: Perceived ease of use
ROC: Receiver operating characteristics
LOT-R: Revised Life-Orientation-Tests
STAI: State-Trait Anxiety Inventory
TRA: Theory of reasoned action
UTAUT: Unified Theory of Acceptance and Use of Technology
