# Supplementary material for: How mental health status and attitudes toward mental health shape AI Acceptance in psychosocial care: a cross-sectional analysis
Source: BMC Psychol. 2025 Jun 6;13:617. doi: 10.1186/s40359-025-02954-z (PMC12143098; doi:10.1186/s40359-025-02954-z)
Supplement: Supplementary file 1 — Supplementary Material 1 [file 40359_2025_2954_MOESM1_ESM.pdf]

Supplement S1 on detailed sociodemographic data

Table S1-1. Participant's additional sociodemographic characteristics not listed in the main text of the article

	N	%
Living Environment		
Village/rural (less than 5,000 inhabitants)	78	25.8
Small town (5,000 to 20,000 inhabitants)	88	29.1
Large city (over 20,000 inhabitants)	136	45.0
Country		
Germany	298	98.7
Austria	2	0.7
Other	2	0.7
Migration Background		
Yes	23	7.6
No	276	91.4
Not specified	3	1.0
Mother tongue		
German	296	98.0
Other/Not specified	6	2.0
Marital status		
Single	84	27.8
In partnership	58	19.2
Married	136	45.0
Widowed	3	1.0
Divorced	16	5.3
Other/Not specified	6	2.0
Children		
0	161	53.3
1	22	7.3
2	54	17.9
≥ 3	54	17.9
Not specified	11	3.6
Income		
< 1500 €	46	15.2
1500€ to under 2600 €	43	14.2
2600€ to under 5000 €	149	49.4
≥ 5000€	54	17.9
Not specified	10	3.3
Education		
Comprehensive school-leaving certificat[1]	5	1.7
Completed vocational training/apprenticeship	8	2.6
Technical college entrance qualification[2]	17	5.6
General higher education entrance qualification[3]	40	13.2
Bachelor's degree	42	13.9

Master's degree	171	56.6
Doctorate (PhD)/Habilitation	19	6.3
Employment		
In training (school, student, apprenticeship)	48	15.8
Employee (incl. civil servant)	213	70.6
Self-employed	11	3.6
Unemployed	4	1.3
Pensioner, retired, in early retirement	14	4.6
Other/ Not specified	12	4.0

Notes: [1]equivalent to completion of lower or intermediate secondary education; [2]Fachhochschulreife; [3]Abitur
