# Supplementary material for: How mental health status and attitudes toward mental health shape AI Acceptance in psychosocial care: a cross-sectional analysis
Source: BMC Psychol. 2025 Jun 6;13:617. doi: 10.1186/s40359-025-02954-z (PMC12143098; doi:10.1186/s40359-025-02954-z)
Supplement: Supplementary file 2 — Supplementary Material 2 [file 40359_2025_2954_MOESM2_ESM.pdf]

Supplement S2. Validation of the questionnaire on AI Aspects (Digital Competence, AI Awareness, Perceived Usefulness of integrating AI into psychosocial care)

The AI aspects 'digital competence and AI awareness' and 'perceived usefulness of integrating AI into psychosocial care' were assessed with eight items, formulated by McLennan et al. (2022) for medical students. The original items were adapted to the general population. As we translated the items into German and due to the adaptation, we validated the questionnaire in our sample. We investigated the factor structure of the AI items by conducting an item analysis, difficulty analysis, and discrimination analysis as well as an exploratory factor analysis (EFA). The best factor solution was explored by parallel analysis, minimum average partial test (MAP) and fit indices [Bayesian Information Criterion (BIC), Root Mean Square Error of Approximation (RMSEA) and Tucker-Lewis Index (TLI)]. Reliability and validity were assessed through Cronbach's Alpha and correlation analyses. Divergent validity was examined with ASKU (Beierlein et al. 2012) and BMI (Nohr et al. 2021; Hirai und Clum 2000), while convergent validity was assessed with the eHEALS (Soellner et al. 2014; Norman et al. 2006).

Item analysis of the AI items showed that none of the items exhibited normal distribution (Kolmogorov-Smirnov and Shapiro-Wilk $p < .001$ for all items). All items utilized the full range and were mostly right-skewed and left-tailed except for item 4 ("support for mental health professionals in making a diagnosis, for example using a diagnostic algorithm"), which was nearly symmetrical. Item 1 ("overall, I have good digital skills and competencies") had the highest statistical difficulty, indicating that a large number of individuals found this item easy to answer. Item 7 ("independent treatment by patients with AI programs (such as ChatGPT) or AI health apps") had the lowest difficulty. All discrimination indices were significant at a level of $p < .001$.

Parallel Analysis indicated a three and MAP-test a one factor solution. Fit Indices were tested for the 1-, 2- and 3-factor solution. The fit indices suggest that the 2-factor solution is superior to the 1-factor solution. The 3-factor solution did not seem to fit the data well, as the goodness-of-fit test is not significant. Based on the analysis, the data suggests a two-factor structure (WLS, promax). Factor 1, comprising 2 Items (#1 and #2) was labelled "AI awareness and digital skills" and Factor 2, comprising 6 items (#3 to #8) was labelled "Perceived usefulness of integrating AI into psychosocial care".

Correlational analyses for convergent and divergent validity showed the expected significant and non-significant correlations between the two factors of the questionnaire on AI aspects and related or distant constructs, respectively. We did not assess constructs related to perceived usefulness so that convergent validation for this factor could not be determined. Correlation coefficients are depicted in the Table S2-1.

Table S2-1. Correlation coefficients for the convergent and divergent validity.

	Information appraisal (eHEALS)	Information seeking (eHEALS)	Self-efficacy (ASKU)	Stigmatization (BMI)
Digital competences/ AI awareness	.502<br>(p <.001)<br>n = 317	.472<br>(p <.001)<br>n = 317	.126<br>(p<.05)<br>n = 317	-.002<br>*ns*<br>n = 306
Perceived Usefulness	.091<br>*ns*<br>n = 317	.096<br>*ns*<br>n = 317	-.027<br>*ns*<br>n = 317	-.062<br>*ns*<br>n = 306
