# Supplementary material for: How mental health status and attitudes toward mental health shape AI Acceptance in psychosocial care: a cross-sectional analysis
Source: BMC Psychol. 2025 Jun 6;13:617. doi: 10.1186/s40359-025-02954-z (PMC12143098; doi:10.1186/s40359-025-02954-z)
Supplement: Supplementary file 3 — Supplementary Material 3 [file 40359_2025_2954_MOESM3_ESM.pdf]

Supplement S3 on regression results

Table S3-1. Variables, excluded from the respective model during the stepwise hierarchical regression analysis predicting the perceived utility of integrating AI into psychosocial care (N = 269). Note that per analysis the first model only includes variables from the first block and that all variables from the first block were forced into the respective regression models.

	β	T	p
**Model 1**			
B2: Neuroticism (BFI-10)	-.089	-1.424	.156
B2: Extraversion (BFI-10)	.111	1.906	.058
B2: Openness (BFI-10)	-.113	-1.948	.052
B2: Conscientiousness (BFI-10)	-.101	-1.759	.080
B2: Agreeableness (BFI-10)	.155	2.722	.007
B2: Pessimism (LOT-R)	-.138	-2.335	.020
B2: Optimism (LOT-R)	.116	1.941	.053
B3: Help seeking (IASMHS)	.103	1.739	.083
B3: Psychological openness (IASMHS)	.027	.452	.651
B3: Help seeking propensity (ISAMHS)	.111	1.838	.067
B3: Indifference to stigma (IASMHS)	.110	1.917	.056
B3: Attitudes and skills (MHLS)	.174	2.955	.003
B3: MHL knowledge (MHLS)	.012	.193	.847
B3: Psychological Distress (K10)	-.126	-1.989	.048
B3: Informal help seeking (GHSQ)	.072	1.259	.209
B3: Formal but non-professional help seeking (GHSQ)	.061	.977	.329
B3: Professional help seeking (GHSQ)	.121	2.015	.045
B3: Mental Health Problems (GHQ-12)	-.040	-.667	.505
B3: Resilience (BRS)	.110	1.743	.083
B3: Self-efficacy (PSS-2&2)	.064	1.090	.277
B3: Helplessness (PSS-2&2)	-.137	-2.330	.021
**Model 2**			
B2: Neuroticism (BFI-10)	-.043	-.661	.509
B2: Extraversion (BFI-10)	.084	1.424	.156
B2: Openness (BFI-10)	-.118	-2.066	.040
B2: Conscientiousness (BFI-10)	-.109	-1.930	.055
B2: Pessimism (LOT-R)	-.102	-1.673	.096
B2: Optimism (LOT-R)	.062	.965	.336
B3: Help seeking (IASMHS)	.077	1.292	.198
B3: Psychological openness (IASMHS)	.004	.073	.942
B3: Help seeking propensity (ISAMHS)	.085	1.388	.166
B3: Indifference to stigma (IASMHS)	.092	1.610	.109
B3: Attitudes and skills (MHLS)	.160	2.724	.007
B3: MHL knowledge (MHLS)	-.005	-.082	.935
B3: Psychological Distress (K10)	-.101	-1.588	.113
B3: Informal help seeking (GHSQ)	.066	1.157	.248
B3: Formal but non-professional help seeking (GHSQ)	.051	.815	.416
B3: Professional help seeking (GHSQ)	.101	1.686	.093
B3: Mental Health Problems (GHQ-12)	-.016	-.268	.789
B3: Resilience (BRS)	.065	.988	.324
B3: Self-efficacy (PSS-2&2)	.028	.472	.637
B3: Helplessness (PSS-2&2)	-.113	-1.926	.055
**Model 3**			
B2: Neuroticism (BFI-10)	-.049	-.757	.450
B2: Extraversion (BFI-10)	.105	1.775	.077
B2: Conscientiousness (BFI-10)	-.099	-1.745	.082

B2: Pessimism (LOT-R)	-.126	-2.063	.040
B2: Optimism (LOT-R)	.082	1.269	.206
B3: Help seeking (IASMHS)	.085	1.442	.151
B3: Psychological openness (IASMHS)	.020	.326	.745
B3: Help seeking propensity (ISAMHS)	.085	1.397	.164
B3: Indifference to stigma (IASMHS)	.097	1.707	.089
B3: Attitudes and skills (MHLS)	.169	2.906	.004
B3: MHL knowledge (MHLS)	.000	.007	.994
B3: Psychological Distress (K10)	-.101	-1.601	.111
B3: Informal help seeking (GHSQ)	.068	1.215	.225
B3: Formal but non-professional help seeking (GHSQ)	.041	.656	.512
B3: Professional help seeking (GHSQ)	.098	1.647	.101
B3: Mental Health Problems (GHQ-12)	-.024	-.401	.689
B3: Resilience (BRS)	.076	1.162	.246
B3: Self-efficacy (PSS-2&2)	.041	.682	.496
B3: Helplessness (PSS-2&2)	-.121	-2.073	.039
**Model 4**			
B2: Neuroticism (BFI-10)	.008	.115	.908
B2: Extraversion (BFI-10)	.071	1.138	.256
B2: Conscientiousness (BFI-10)	-.130	-2.265	.024
B2: Optimism (LOT-R)	.019	.247	.805
B3: Help seeking (IASMHS)	.052	.830	.407
B3: Psychological openness (IASMHS)	-.024	-.382	.703
B3: Help seeking propensity (ISAMHS)	.064	1.036	.301
B3: Indifference to stigma (IASMHS)	.072	1.218	.224
B3: Attitudes and skills (MHLS)	.152	2.575	.011
B3: MHL knowledge (MHLS)	.003	.052	.959
B3: Psychological Distress (K10)	-.039	-.511	.610
B3: Informal help seeking (GHSQ)	.051	.904	.367
B3: Formal but non-professional help seeking (GHSQ)	.047	.757	.450
B3: Professional help seeking (GHSQ)	.093	1.566	.119
B3: Mental Health Problems (GHQ-12)	.042	.629	.530
B3: Resilience (BRS)	.017	.231	.818
B3: Self-efficacy (PSS-2&2)	-.027	-.398	.691
B3: Helplessness (PSS-2&2)	-.088	-1.388	.166
**Model 5**			
B2: Neuroticism (BFI-10)	.006	.092	.092
B2: Extraversion (BFI-10)	.077	1.243	1.243
B2: Optimism (LOT-R)	.035	.464	.464
B3: Help seeking (IASMHS)	.057	.925	.925
B3: Psychological openness (IASMHS)	-.017	-.269	-.269
B3: Help seeking propensity (ISAMHS)	.066	1.077	1.077
B3: Indifference to stigma (IASMHS)	.075	1.291	1.291
B3: Attitudes and skills (MHLS)	.167	2.846	2.846
B3: MHL knowledge (MHLS)	.016	.270	.270
B3: Psychological Distress (K10)	-.059	-.772	-.772
B3: Informal help seeking (GHSQ)	.059	1.050	1.050
B3: Formal but non-professional help seeking (GHSQ)	.038	.617	.617
B3: Professional help seeking (GHSQ)	.117	1.971	1.971
B3: Mental Health Problems (GHQ-12)	.027	.406	.406
B3: Resilience (BRS)	.035	.482	.482
B3: Self-efficacy (PSS-2&2)	-.012	-.171	-.171
B3: Helplessness (PSS-2&2)	-.098	-1.557	-1.557

**Model 6**			
B2: Neuroticism (BFI-10)	.009	.128	.898
B2: Extraversion (BFI-10)	.071	1.152	.251
B2: Optimism (LOT-R)	.033	.451	.652
B3: Help seeking (IASMHS)	-.117	-1.401	.163
B3: Psychological openness (IASMHS)	-.148	-2.065	.040
B3: Help seeking propensity (ISAMHS)	-.046	-.627	.531
B3: Indifference to stigma (IASMHS)	-.026	-.371	.711
B3: MHL knowledge (MHLS)	-.041	-.655	.513
B3: Psychological Distress (K10)	-.050	-.657	.512
B3: Informal help seeking (GHSQ)	.016	.284	.777
B3: Formal but non-professional help seeking (GHSQ)	.020	.330	.742
B3: Professional help seeking (GHSQ)	.061	.945	.346
B3: Mental Health Problems (GHQ-12)	.030	.456	.649
B3: Resilience (BRS)	.015	.203	.839
B3: Self-efficacy (PSS-2&2)	-.043	-.626	.532
B3: Helplessness (PSS-2&2)	-.089	-1.437	.152
**Model 7**			
B2: Neuroticism (BFI-10)	.023	.326	.745
B2: Extraversion (BFI-10)	.071	1.167	.244
B2: Optimism (LOT-R)	.001	.013	.990
B3: Help seeking (IASMHS)	-.002	-.019	.985
B3: Help seeking propensity (ISAMHS)	-.038	-.520	.604
B3: Indifference to stigma (IASMHS)	.030	.401	.688
B3: MHL knowledge (MHLS)	-.043	-.694	.488
B3: Psychological Distress (K10)	-.055	-.722	.471
B3: Informal help seeking (GHSQ)	.023	.401	.689
B3: Formal but non-professional help seeking (GHSQ)	.014	.234	.815
B3: Professional help seeking (GHSQ)	.064	.997	.320
B3: Mental Health Problems (GHQ-12)	.018	.277	.782
B3: Resilience (BRS)	.007	.098	.922
B3: Self-efficacy (PSS-2&2)	-.059	-.863	.389
B3: Helplessness (PSS-2&2)	-.103	-1.660	.098

Notes. B2/3=Block 2/3; β, standardized beta coefficient.

Table S3-2. Variables, excluded from the respective model during the stepwise hierarchical regression analysis predicting the actual usage of mental health apps (N = 276). Note that per analysis the first model only includes variables from the first block and that all variables from the first block were forced into the respective regression models.

	β	T	p
**Model 1**			
B2: Neuroticism (BFI-10)	.009	141	.888
B2: Extraversion (BFI-10)	-.065	-1.113	.267
B2: Openness (BFI-10)	-.085	-1.470	.143
B2: Conscientiousness (BFI-10)	-.091	-1.583	.115
B2: Agreeableness (BFI-10)	.018	.310	.757
B2: Pessimism (LOT-R)	.088	1.487	.138
B2: Optimism (LOT-R)	-.086	-1.446	.149
B3: Help seeking (IASMHS)	.026	.437	.663
B3: Psychological openness (IASMHS)	-.030	-.500	.617
B3: Help seeking propensity (ISAMHS)	.052	.862	.389
B3: Indifference to stigma (IASMHS)	.039	.679	.498
B3: Attitudes and skills (MHLS)	.029	.483	.629
B3: MHL knowledge (MHLS)	.082	1.337	.182
B3: Psychological Distress (K10)	.209	3.342	.001
B3: Informal help seeking (GHSQ)	-.116	-2.041	.042
B3: Formal but non-professional help seeking (GHSQ)	.178	2.887	.004
B3: Professional help seeking (GHSQ)	.077	1.285	.200
B3: Mental Health Problems (GHQ-12)	.103	1.719	.087
B3: Resilience (BRS)	-.019	-.293	.770
B3: Self-efficacy (PSS-2&2)	-.023	-.383	.702
B3: Helplessness (PSS-2&2)	.005	.085	.932
**Model 2**			
B2: Neuroticism (BFI-10)	-.135	-1.884	.061
B2: Extraversion (BFI-10)	.007	.112	.911
B2: Openness (BFI-10)	-.086	-1.507	.133
B2: Conscientiousness (BFI-10)	-.053	-.912	.363
B2: Agreeableness (BFI-10)	.050	.861	.390
B2: Pessimism (LOT-R)	-.036	-.510	.611
B2: Optimism (LOT-R)	.011	.162	.872
B3: Help seeking (IASMHS)	.100	1.627	.105
B3: Psychological openness (IASMHS)	.021	.348	.728
B3: Help seeking propensity (ISAMHS)	.109	1.771	.078
B3: Indifference to stigma (IASMHS)	.104	1.758	.080
B3: Attitudes and skills (MHLS)	.064	1.074	.284
B3: MHL knowledge (MHLS)	.077	1.271	.205
B3: Informal help seeking (GHSQ)	-.088	-1.554	.121
B3: Formal but non-professional help seeking (GHSQ)	.182	3.001	.003
B3: Professional help seeking (GHSQ)	.121	2.010	.045
B3: Mental Health Problems (GHQ-12)	-.087	-1.008	.315
B3: Resilience (BRS)	.114	1.588	.113
B3: Self-efficacy (PSS-2&2)	.140	1.962	.051
B3: Helplessness (PSS-2&2)	-.124	-1.850	.065
**Model 3**			
B2: Neuroticism (BFI-10)	-.142	-2.008	046
B2: Extraversion (BFI-10)	-.018	-.299	.765
B2: Openness (BFI-10)	-.076	-1.351	.178
B2: Conscientiousness (BFI-10)	-.040	-.706	.481

B2: Agreeableness (BFI-10)	.038	.665	.507
B2: Pessimism (LOT-R)	-.052	-.740	.460
B2: Optimism (LOT-R)	.006	.091	.928
B3: Help seeking (IASMHS)	.095	1.569	.118
B3: Psychological openness (IASMHS)	.026	.427	.669
B3: Help seeking propensity (ISAMHS)	.088	1.450	.148
B3: Indifference to stigma (IASMHS)	.106	1.826	.069
B3: Attitudes and skills (MHLS)	.050	.859	.391
B3: MHL knowledge (MHLS)	.068	1.132	.259
B3: Informal help seeking (GHSQ)	-.108	-1.916	.056
B3: Professional help seeking (GHSQ)	.072	1.148	.252
B3: Mental Health Problems (GHQ-12)	-.065	-.765	.445
B3: Resilience (BRS)	.105	1.487	.138
B3: Self-efficacy (PSS-2&2)	.123	1.750	.081
B3: Helplessness (PSS-2&2)	-.125	-1.895	.059

Notes. B2/3=Block 2/3; β, standardized beta coefficient.
