# Supplementary material for: How mental health status and attitudes toward mental health shape AI Acceptance in psychosocial care: a cross-sectional analysis
Source: BMC Psychol. 2025 Jun 6;13:617. doi: 10.1186/s40359-025-02954-z (PMC12143098; doi:10.1186/s40359-025-02954-z)
Supplement: Supplementary file 4 — Supplementary Material 4 [file 40359_2025_2954_MOESM4_ESM.pdf]

Supplement S4. German items measuring digital competence, AI awareness and perceived utility of integrating AI into psychosocial care

Im Folgenden geht es um Ihre Einschätzung zum Einsatz von Künstlicher Intelligenz im Rahmen von Psychotherapie und psychischer Gesundheit.

Jeden Tag nutzen wir sogenannte Künstliche Intelligenz oder kurz KI. Meistens ohne, dass wir es merken. Wenn wir auf YouTube nach Videos, bei Spotify nach Musik oder bei Google nach Informationen suchen oder Siri und Alexa nutzen, spielt KI immer eine wichtige Rolle. So wie wir Menschen, sind KI-Systeme in der Lage, auf Grundlage von Informationen Entscheidungen zu treffen. Ähnlich wie wir können KI-Systeme über die Zeit dazulernen. Dafür benötigt die KI möglichst viele Informationen. Je mehr Informationen eine KI erhält, desto besser kann sie die an sie gestellten Aufgaben und Probleme lösen und Entscheidungen treffen.

Bitte lesen Sie die folgenden Aussagen und geben auf der Skala an, inwiefern Sie den Aussagen zustimmen oder sie ablehnen. Es gibt dabei keine richtigen oder falschen Antworten.

1. Ich habe insgesamt gute digitale Fähigkeiten und Kompetenzen.
    - 1- Stimme überhaupt nicht zu 2- Stimme eher nicht zu 3- neutral 4-Stimme eher zu 5-Stimme vollkommen zu - (-1) keine Angabe
2. Ich fühle mich über künstliche Intelligenz (KI) im Zusammenhang mit psychischer Gesundheit informiert.
    - 1- Stimme überhaupt nicht zu 2- Stimme eher nicht zu 3- neutral 4-Stimme eher zu 5-Stimme vollkommen zu - (-1) keine Angabe
3. Künstliche Intelligenz kann im Bereich der psychischen Gesundheit nützlich sein.
    - 1- Stimme überhaupt nicht zu 2- Stimme eher nicht zu 3- neutral 4-Stimme eher zu 5-Stimme vollkommen zu
4. Unterstützung von psychosozialen Fachkräften bei der Erstellung einer Diagnose, etwa per Diagnose-Algorithmus.
    - 1- überhaupt nicht nützlich 2- eher nicht nützlich 3- neutral 4-eher nützlich 5-vollkommen nützlich
5. Unterstützung von psychosozialen Fachkräften bei Empfehlungen oder Behandlungsentscheidungen, etwa durch Algorithmus-Vorschläge für Behandlungsoptionen.
    - 1- überhaupt nicht nützlich 2- eher nicht nützlich 3- neutral 4-eher nützlich 5-vollkommen nützlich
6. Unterstützung von psychosozialen Fachkräften direkt bei der Beratung oder Behandlung, etwa durch KI-gestützte Virtual Reality (VR) Anwendungen.
    - 1- überhaupt nicht nützlich 2- eher nicht nützlich 3- neutral 4-eher nützlich 5-vollkommen nützlich
7. Die selbständige Behandlung durch PatientInnen mit KI-Programmen (etwa ChatGPT) oder KI-Gesundheits-Apps.
    - 1- überhaupt nicht nützlich 2- eher nicht nützlich 3- neutral 4-eher nützlich 5-vollkommen nützlich
8. Denken Sie, dass künstliche Intelligenz bei der Verbesserung Ihrer psychischen Gesundheit sinnvoll eingesetzt werden kann?
    - 1 - überhaupt nicht 2 - eher nicht 3 - teilweise 4 - eher 5 – sehr - (-1) Keine Angabe

Gesundheits-Apps

9. Nutzen Sie Gesundheits-Apps (z.B. zur Entspannung, zum Stressabbau oder zur (unterstützenden) Behandlung psychischer Störungen)?
   - 1 – Nie 2 - Selten 3 – Gelegentlich 4 – Oft 5 - Immer
10. Wie viele Gesundheits-Apps haben Sie aktuell auf Ihrem Smartphone installiert?
    - Numerische Angabe 0 bis 15
11. Bezogen auf alle Gesundheits-Apps, die Sie bisher verwendet haben, wie häufig haben Sie die folgenden Hilfsangebote/Inhalte genutzt?
    - 1 – Nie 2 - Selten 3 – Gelegentlich 4 – Oft 5 – Immer

    a. Entspannungs-, Meditations- und Achtsamkeitsübungen
    b. Unterstützung beim Stressmanagement
    c. Hilfe bei der Tagesstruktur/beim Zeitmanagement
    d. Möglichkeit, sich mit anderen über die psychische Gesundheit auszutauschen (online Plattformen/Foren)
    e. Unterstützung bei Schlafdauer bzw. Schlafqualität
    f. Aufschreiben von Gefühlen, Gedanken oder Aktivitäten (z.B. digitales Tagebuch)
    g. zur (unterstützenden) Behandlung psychischer Störungen (bspw. bei Depressionen, Ängsten etc.)
